# Use of Mood- and Stress-Relief Dietary Supplements and Over-the-Counter Medications in Relation to Physician Trust, Hypothetical Help-Seeking Preferences, and Communication-Related Attitudes: A Cross-Sectional Survey

**DOI:** 10.3390/nu18162726

**Published:** 2026-08-20

**Authors:** Aleksandra Brzozowska, Jakub Grabowski, Maciej Brosz

**Affiliations:** 1 Adult Psychiatry Scientific Circle, Division of Developmental Psychiatry, Psychotic and Geriatric Disorders, Medical University of Gdansk, 80-282 Gdansk, Poland; 2 Division of Developmental Psychiatry, Psychotic and Geriatric Disorders, Medical University of Gdansk, 80-282 Gdansk, Poland; 3 Institute of Sociology, Faculty of Social Sciences, University of Gdansk, 80-309 Gdansk, Poland; maciej.brosz@ug.edu.pl

**Keywords:** dietary supplements, over-the-counter medications, psychological distress, stress management, trust in physicians, coping strategies, nutritional supplements, anxiety

## Abstract

**Background/Objectives:** Dietary supplements (DSs) and over-the-counter (OTC) medications for mood improvement and stress relief are widely used, but little is known about how their use relates to coping preferences, trust in physicians, and communication with doctors. This exploratory cross-sectional study compared users and non-users of such products in Poland. **Methods:** Data were collected between October 2024 and May 2025 and included 798 adults recruited through a mixed-mode convenience sampling strategy (636 online and 162 in person). Participants were classified as current users (CUs; *n* = 184), past users (PUs; *n* = 297), or never-users (NUs; *n* = 317). The outcomes included hypothetical future coping strategies (under an assumption of unlimited resources), trust in physicians using an author-translated adaptation of the Trust in Doctors in General (T-DiG) scale and three-category communication-related attitudes. Multivariable models were adjusted for age, gender, education, medical or health science background, and recruitment mode. **Results:** Overall, 60.3% of respondents reported current or past use. Compared with NUs, both CUs and PUs had higher adjusted odds of selecting future DS/OTC use (CUs: aOR = 5.60, 95% CI 3.44–9.14, *p* < 0.001; PUs: aOR = 2.44, 95% CI 1.52–3.93, *p* < 0.001). PUs also had higher adjusted odds of selecting psychotherapy (aOR = 1.50, 95% CI 1.04–2.17, *p* = 0.031; overall *p* = 0.018). Product-use status was associated with selected items of T-DiG: communication competency (overall *p* = 0.031) and global trust (overall *p* < 0.001). Compared with NUs, CUs had lower adjusted scores on both subscales (aMD = −0.19, 95% CI −0.33 to −0.04, *p* = 0.010 and aMD = −0.40, 95% CI −0.59 to −0.21, *p* < 0.001, respectively). Overall product-group effects were also observed for the first two communication items: CUs had lower relative risk ratios than NUs for discussing product-related doubts first with their physician (RRR = 0.52, 95% CI 0.35–0.78, *p* = 0.001) and for feeling free to raise such doubts (RRR = 0.57, 95% CI 0.36–0.92, *p* = 0.022). **Conclusions:** Product-use status showed selective associations with hypothetical coping preferences, physician trust, and communication-related attitudes rather than a uniform pattern of reluctance to seek professional support. Current use was most strongly associated with lower trust and less favorable product-related communication attitudes. Given the cross-sectional design and hypothetical nature of the coping outcomes, these findings do not establish causality, temporal ordering, or actual healthcare-seeking behavior.

## 1. Introduction

Depressed mood and stress-related symptoms are common mental health complaints that do not necessarily indicate the presence of a mental disorder. They may refer to a wide range of self-perceived emotional, cognitive, somatic, and behavioral difficulties, such as tension, irritability, worry, fatigue, sleep problems, sadness, emotional exhaustion, reduced motivation, or a perceived inability to cope with daily demands [1,2,3,4]. These experiences are often transient and subclinical. However, when persistent, severe, or associated with significant distress and functional impairment, they may overlap with or develop into clinically significant mental health conditions, including depressive, anxiety, adjustment, or other stress-related disorders [5,6]. These phenomena are often understood as dimensional constructs that range from everyday psychological distress to symptom patterns meeting formal diagnostic criteria [7,8]. They represent a substantial and growing burden on mental health, affecting a significant proportion of the general population, and may be associated with impaired functioning and reduced quality of life [9,10,11].

There are various ways to cope with stress and depressed mood. Formal approaches primarily include psychiatric care and psychotherapy, while informal strategies often rely on social support, such as talking to family members or close friends [12]. Some individuals engage in complementary and alternative medicine (CAM) practices, including the use of dietary supplements (DSs) and over-the-counter (OTC) medications for mood improvement or stress relief [13,14,15]. The popularity of such preparations has increased in recent years. They are widely available on the European market (including in Poland), and their market value continues to grow annually [16,17,18]. Commonly used ingredients include *Withania somnifera* (ashwagandha), *Melissa officinalis*, *Valeriana officinalis*, *Humulus lupulus* (hops), *Hypericum perforatum* (St. John’s wort), or cannabidiol [13,19,20]. This trend is likely driven by their easy accessibility, extensive marketing, and the widespread perception that “natural” products are inherently safe [21,22]. Contrary to popular belief, the use of these products is not without risk. Certain ingredients may interact with prescribed medications, potentially compromising the treatment of chronic conditions and exposing patients to adverse health outcomes [23,24,25,26]. These concerns are further amplified by the relatively limited regulation of the DS market in Poland and other European countries, raising questions about product quality and patient safety [27,28,29,30]. Given that these products are frequently selected without medical supervision, their use also raises questions about whether and under what conditions patients involve physicians in decisions about self-management.

In this context, trust in physicians may be particularly relevant. Trust is a key determinant of health-related behaviors: it has been associated with subjective health outcomes, treatment adherence, patient satisfaction, and symptom severity [31,32,33]. Weber et al. identified ten domains of trust in physicians, i.e., fidelity, technical competence, communicative competence, interpersonal competence, honesty, confidentiality, global, behavioral, fairness, and system trust/accountability [34]. Instruments such as Trust in My Doctor (T-MD) and Trust in Doctors in General (T-DiG) have been developed to capture some of these dimensions, enabling a more nuanced assessment of patient trust [35].

The inclusion of communicative competence among the domains of trust underscores the close relationship between patient trust and patient–physician communication. Empirical studies further indicate that patients’ perceptions of physicians’ communication are associated with higher levels of trust [34,35,36]. This connection is clinically relevant because trust and effective communication may facilitate the disclosure of health-related behaviors that patients do not always report spontaneously. This relationship is particularly important in the context of DSs and OTC medications, as patients often do not disclose their use of such products to physicians [37,38]. In the context of products used for mood improvement or stress relief, communication may be relevant at two levels: not only whether patients disclose product use, but also whether they feel able to discuss the underlying mental health concerns that prompted such use.

The present study was a secondary analysis of the same survey dataset previously used to examine the prevalence and patterns of DSs and OTC medication used for mood improvement and stress relief [39]. The companion publication focused primarily on prevalence, product-use patterns, factors influencing product choice, safety-related practices, and disclosure of product use to physicians. In contrast, the present analysis focuses on physician trust domains, hypothetical future help-seeking and self-management preferences, and communication-related attitudes.

To our knowledge, these issues have not been systematically investigated in the context of mood- and stress-relief preparations. Existing evidence provides reasons to expect that users and non-users may differ in their attitudes and coping preferences. Research on CAM suggests that lower trust in conventional healthcare or physicians may be associated with greater reliance on non-conventional health products and practices, although the evidence remains heterogeneous and has not specifically focused on products used for mood improvement or stress relief [40,41,42]. In addition, psychological distress is often managed through strategies outside formal healthcare, including informal support and self-management [15,19,40]. Accordingly, it was hypothesized that users would be more likely to rely on informal coping strategies (H1), exhibit lower levels of trust in physicians (H2), and be less willing to discuss their potential use of such products and mental health problems with their doctor (H3). Examining these relationships may help improve the quality of care for individuals experiencing depressed mood and stress-related difficulties.

H1–H3 were formulated before the statistical analyses reported in the present manuscript. Because the study was exploratory and included author-developed and non-validated translated measures, the findings are interpreted as hypothesis-generating rather than confirmatory.

## 2. Materials and Methods

### 2.1. Study Design

This exploratory cross-sectional study was a secondary analysis of the survey dataset previously reported in the companion publication [39]. It used a Polish-language questionnaire and examined physician trust, hypothetical help-seeking and self-management preferences, and communication-related attitudes, which were not the primary outcomes of the companion analysis. Data collection took place from 8 October 2024 to 6 May 2025. A non-probability convenience sampling strategy combined online and in-person recruitment. No quotas or random sampling procedures were used.

### 2.2. Participants, Recruitment, and Eligibility

Online questionnaires were distributed through open recruitment on Facebook and Instagram and through institutional mail to students and employees of the Medical University of Gdańsk. In-person recruitment was conducted by four trained interviewers who approached adult passers-by on the streets of Gdańsk, Sopot, and Gdynia. The same Google Forms questionnaire was used in both modes: online respondents completed it independently, whereas in-person responses were read aloud, recorded by the interviewer, and entered directly into Google Forms. Interviewers marked the designated interviewer-initials field for these entries, which was used to identify recruitment mode. Records containing interviewer initials classified as in person and all remaining records as online. This coding rule yielded 162 in-person and 636 online records, and the corrected recruitment-mode variable was used consistently in descriptive comparisons and all adjusted models. The numbers of individuals who viewed the online invitation or were approached in person but did not submit the questionnaire were not recorded; therefore, a response rate cannot be calculated.

**Figure 1:**
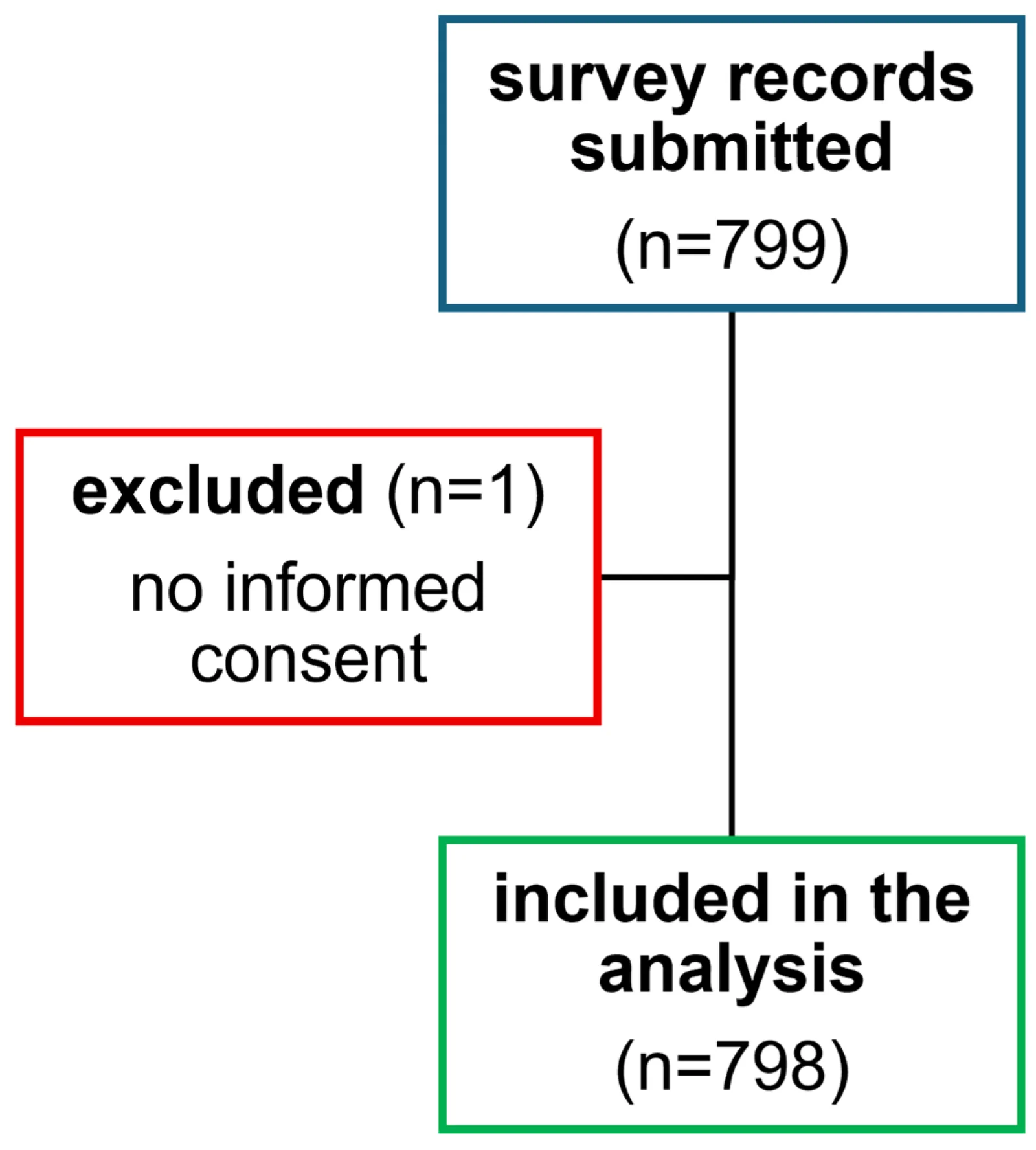
Participant disposition flowchart.

The inclusion criteria were age ≥ 18 years, informed consent, sufficient knowledge of Polish to understand the study information and complete the questionnaire, and internet access in the online survey variant. Ability to understand and complete the questionnaire was an operational eligibility requirement. Nationality and place of residence were not collected. The only exclusion criterion was refusal to participate at any stage. Of 799 submitted survey records, one record indicated lack of informed consent and contained only screening information—it was excluded. The remaining 798 consenting records met the analytic eligibility criteria, and no other responses were excluded. The participant flowchart is presented in Figure 1.

Participation was voluntary and anonymous. Informed consent was obtained from all participants before survey initiation. The study was conducted in accordance with the Declaration of Helsinki. The original protocol was approved by the Institutional Ethics Committee of the Medical University of Gdańsk (KB/570/2023, 20 October 2023), before recruitment began on 8 October 2024. The subsequent approval, KB/570-515/2024 (26 November 2024), represented an administrative extension of the previous approval to allow continuation of the study. It did not constitute an amendment to the study procedures and introduced no changes to the recruitment process, questionnaire content, study protocol, or data-collection procedures.

No a priori sample-size calculation was performed because the study was exploratory. Recruitment continued until the prespecified end of the data-collection period (6 May 2025), and the final sample size was determined by the number of eligible consenting responses obtained during this period.

### 2.3. Questionnaire and Variables

The questionnaire was developed de novo in Polish by the research team for this exploratory study because no existing instrument addressed the exact combination of mood- and stress-relief product use, coping preferences, physician trust, and communication-related attitudes. The questionnaire was reviewed by the study supervisor and by experts of the Institutional Ethics Committee of the Medical University of Gdańsk as part of the ethics review process. It consisted of closed-ended questions, some of which allowed multiple responses and/or the addition of a free-text answer.

The initial section collected sociodemographic data, including gender, age, education, and medical or health science background. Because data were collected using two recruitment modes, online and in person, additional analyses were conducted to compare these subsamples with respect to sociodemographic characteristics and product-use status. All responses were collected in a single Google Forms database. No pilot study was conducted.

The primary independent variable was product-use status. Current use reflected participants’ self-classification as currently using a qualifying product. Past use indicated previous but not current use, without a minimum duration or frequency threshold. Participants were classified as current users (CUs) if they reported currently using such products, past users (PUs) if they reported previous, but not current use, and never-users (NUs) if they reported never having used such products. Products encompassed DSs and OTC medications. Subsequent questionnaire items depended on group assignment.

One set of dependent variables comprised preferred future coping strategies. Participants were asked which strategies they would choose to manage prolonged low mood or negative effects of stress if they had unlimited resources. Multiple responses were permitted. Options included psychotherapy, psychiatric consultation, conversation with a close person, use of a DS or OTC medication for mood improvement or stress relief, and consultation with a general practitioner (GP). Each option was analyzed as a separate binary outcome. These variables represent stated hypothetical preferences and not observed help-seeking behavior.

Trust in physicians was assessed using items from the T-DiG scale [35]. The items were translated into Polish by the authors for the purpose of this study. The translation has not undergone formal linguistic, cultural, or psychometric validation; therefore, all trust-related analyses are exploratory. At the time of the study, no validated Polish instrument specifically assessing trust in physicians was available. The list of items used in the study, together with their Polish translations and assigned subscales, is provided in Table A1. Responses used a 5-point scale ranging from 1 to 5. Negatively worded items were reverse-coded before calculating subscale scores such that higher scores indicated higher trust. Mean scores were calculated for each subscale. Subscale scores were calculated only for participants with complete responses to all items of the subscale. Higher scores indicate greater trust, and for stigma-based discrimination, higher scores indicate less perceived stigma-based discrimination. Internal consistency was assessed using Cronbach’s alpha, which was interpreted as reliability evidence only and not as evidence of construct validity.

The remaining dependent variables were author-developed items concerning willingness and perceived ability to discuss potential use of DSs or OTC medications for mood improvement or stress relief and current mental health difficulties with their doctor. Response options were “yes,” “no,” and “no opinion.” These items assessed stated communication-related attitudes rather than observed disclosure behavior. The term “my doctor” was not further operationalized, and the study did not assess whether respondents had a regular physician, recent physician contact, or which type of physician they had in mind.

The control variables used in adjusted models were age, gender, education, medical or health science background, and recruitment mode. Thus, the primary exposure was three-level product-use status, and the dependent variables were the binary coping preferences, seven T-DiG subscale scores, and three-category communication responses.

### 2.4. Statistical Analysis

Descriptive and initial nonparametric analyses were performed using R 4.5.1 (R Foundation for Statistical Computing, Vienna, Austria) and the RStudio environment 2025.05 (Posit team, Boston, MA, USA). Associations between product-use status and categorical variables were assessed using Pearson’s χ^2^ tests, with Cramér’s V reported as a measure of effect size. Because the question on preferred future coping strategies allowed multiple responses, each option was analyzed separately as a dichotomous variable. Trust scores were compared using Kruskal–Wallis H values. Statistical significance was set at *p* < 0.05.

Multivariable analyses were performed in Python 3.12.13 using pandas 2.2.3, NumPy 2.3.5, SciPy 1.17.0, and scikit-learn 1.8.0. NUs served as the reference product-use group. All models included an intercept and were adjusted for age (18–24, 25–39, 40–59, or ≥60 years), gender (female vs. non-female or undisclosed), education (higher vs. below higher education), medical or health science background (yes/no), and recruitment mode (online/in person). The non-female or undisclosed category comprised 149 male participants and six participants who selected another gender or did not disclose their gender. Categories were collapsed to reduce sparse-cell instability while retaining all participants.

Binary hypothetical coping outcomes were analyzed using unpenalized logistic regression with scikit-learn’s LogisticRegression and are reported as adjusted odds ratios (aORs). T-DiG subscale scores were analyzed using ordinary least-squares regression fitted with numpy.linalg.lstsq and HC3 heteroscedasticity-robust standard errors. The product-group coefficients represent adjusted mean differences (aMDs) relative to NUs. Three-category communication outcomes were analyzed using multinomial logistic regression fitted by maximum likelihood with scipy.optimize.minimize. “No” served as the response reference category, and results are reported as relative risk ratios (RRRs). All estimates are presented with 95% confidence intervals. Overall *p*-values for product-use status were calculated using likelihood-ratio tests for the binary and multinomial logistic models and HC3-robust joint Wald tests for the linear models. No penalization or regularization was applied. No data were imputed. T-DiG subscales were analyzed for participants with complete responses to all items forming the relevant subscale, whereas communication analyses used available responses for each item. All tests were two-sided, with *p* < 0.05 considered statistically significant. Given the exploratory nature of the study, no adjustment for multiple testing was applied.

## 3. Results

The study sample consisted of 798 participants. Most respondents were female (*n* = 643; 80.58%), followed by male respondents (*n* = 149; 18.67%). The remaining six participants (0.75%) identified with a gender other than male or female or declined to provide this information. Overall, 481 respondents (60.28%) reported lifetime use of DSs or OTC medications for mood improvement or stress relief. This group included 184 CUs (23.06%) and 297 PUs (37.22%). NUs accounted for 317 respondents (39.72%). Basic sociodemographic characteristics are presented in Table 1.

Of the 798 analyzed participants, 636 were recruited online and 162 in person. Recruitment modes differed in gender, age, education, medical or health science background, and product-use status (all *p* < 0.001; Cramér’s V = 0.139–0.570), with the largest difference observed for age. Online respondents were more often female, younger, and more likely to have a medical or health science background, whereas in-person respondents were older and more often reported never having used DSs or OTC medications for mood improvement or stress relief. Detailed results are presented in Table A2.

The reported product categories were heterogeneous. Among CUs (*n* = 184), 126 (68.5%) reported DSs only, 22 (12.0%) OTC medication only, 19 (10.3%) both, and 17 (9.2%) included a “do not know” response. Because selections could overlap, 147 (79.9%) reported a DS and 41 (22.3%) an OTC medication. Among PUs (*n* = 297), 150 (50.5%) reported DSs only, 54 (18.2%) OTC medication only, 49 (16.5%) both, and 44 (14.8%) included “do not know.” In sum, 200 (67.3%) reported DSs and 104 (35.0%) OTC medication. The ingredient lists included botanical products such as *W. somnifera* (ashwagandha), *M. officinalis*, *V. officinalis*, *H. lupulus* (hops), and *H. perforatum* (St. John’s wort), but herbal preparations were not a separate product-type category.

### 3.1. Preferred Future Coping Strategies

**Figure 2:**
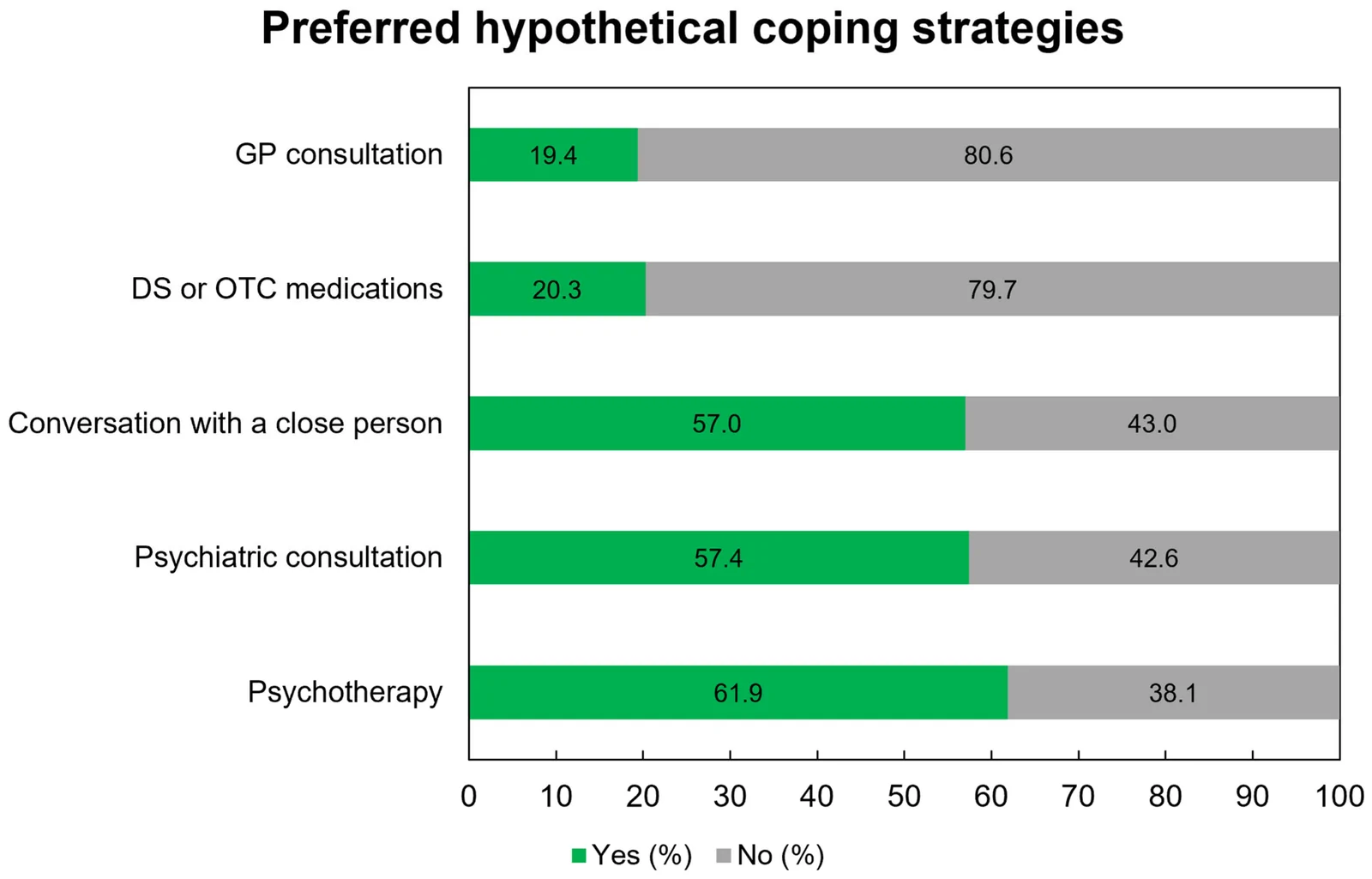
Preferred hypothetical future coping strategies among all participants under the assumption of unlimited financial and time resources.

Participants most often selected psychotherapy (61.9%), psychiatric consultation (57.4%), and conversation with a close person (57.0%) as hypothetical future strategies under the assumption of unlimited resources, while 20.3% selected future use of DSs or OTC medication and 19.4% selected GP consultation (Figure 2).

In unadjusted three-group comparisons, product-use status was associated with psychotherapy, psychiatric consultation, conversation with a close person, and future use of a mood- or stress-relief preparation, but not GP consultation (Table 2). Effect sizes were small, with the largest association for future product use (Cramér’s V = 0.236). CUs and PUs did not differ significantly in past professional mental health support (Table A3).

**Figure 3:**
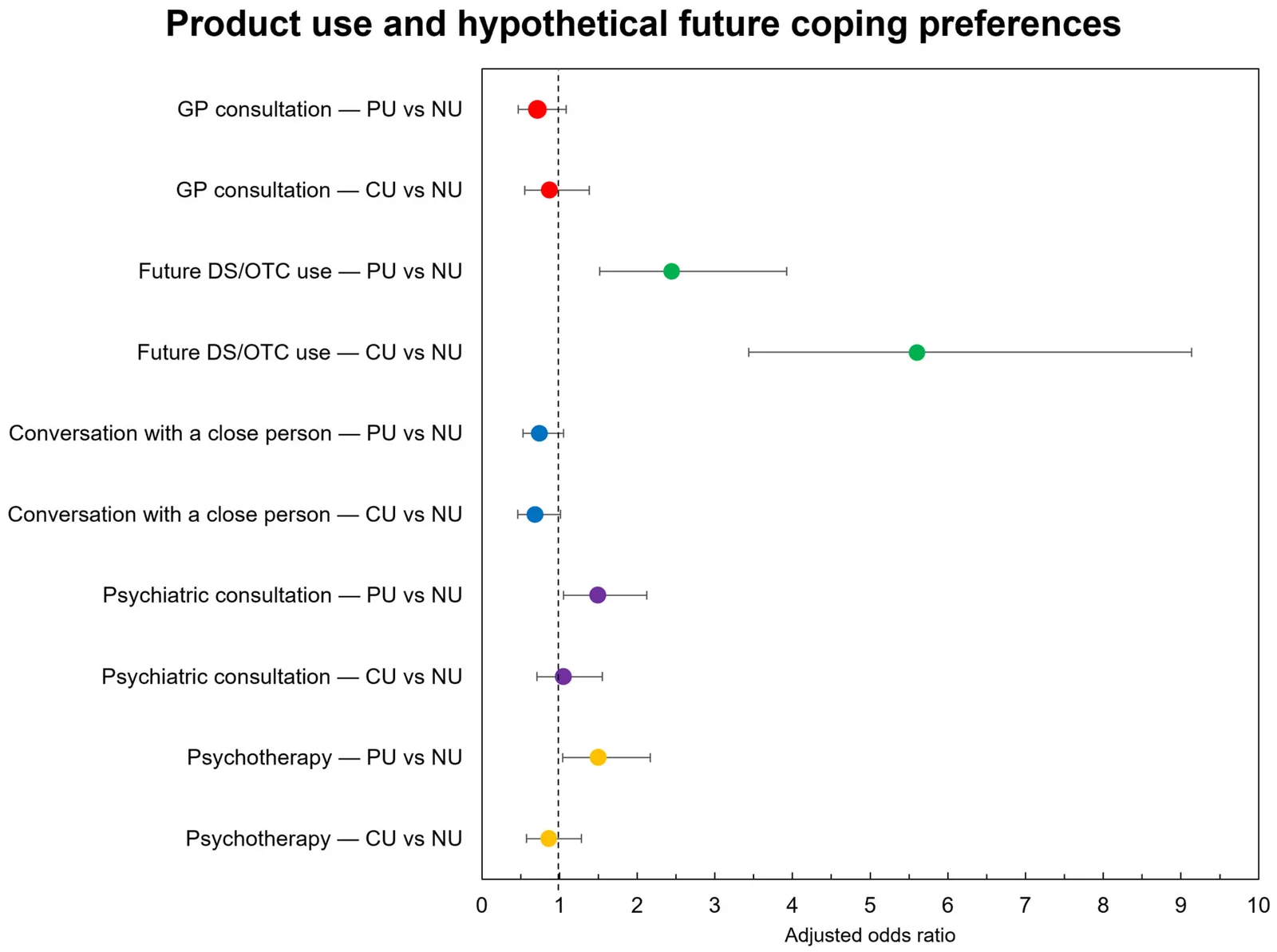
Adjusted odds ratios (aORs) and 95% confidence intervals for hypothetical future coping preferences according to product-use status. NUs served as the reference group. CUs—current users; PUs—past users; NUs—never-users; DSs—dietary supplements; OTC—over-the-counter medications; GP—general practitioner. Different colors added for better differentiation of the analyzed categories.

After covariate adjustment, the overall group effect remained significant for psychotherapy (*p* = 0.018) and future use of a mood- or stress-relief preparation (*p* < 0.001), but not for psychiatric consultation (*p* = 0.057), conversation with a close person (*p* = 0.105), or GP consultation (*p* = 0.285). Compared with NUs, PUs had higher adjusted odds of selecting psychotherapy (aOR = 1.50, 95% CI 1.04–2.17; *p* = 0.031), whereas CUs did not (aOR = 0.86, 95% CI 0.57–1.28; *p* = 0.457). Although the overall group effect for psychiatric consultation did not reach statistical significance (*p* = 0.057), PUs had nominally higher adjusted odds of selecting psychiatric consultation than NUs (aOR = 1.50, 95% CI 1.05–2.12; *p* = 0.024). Future product use was more strongly associated with both CUs (aOR = 5.60, 95% CI 3.44–9.14; *p* < 0.001) and PUs (aOR = 2.44, 95% CI 1.52–3.93; *p* < 0.001) than with NUs. These *p*-values are nominal and are interpreted in the context of the exploratory analysis. The results are presented in Table 3 and Figure 3.

### 3.2. Trust in Physicians

Internal consistency of the author-translated T-DiG subscales ranged from 0.79 to 0.96 in available subscale data (Table 4).

Unadjusted three-group comparisons identified significant differences for communication competency and global trust, whereas the remaining subscales were not statistically significant (Table 5).

**Figure 4:**
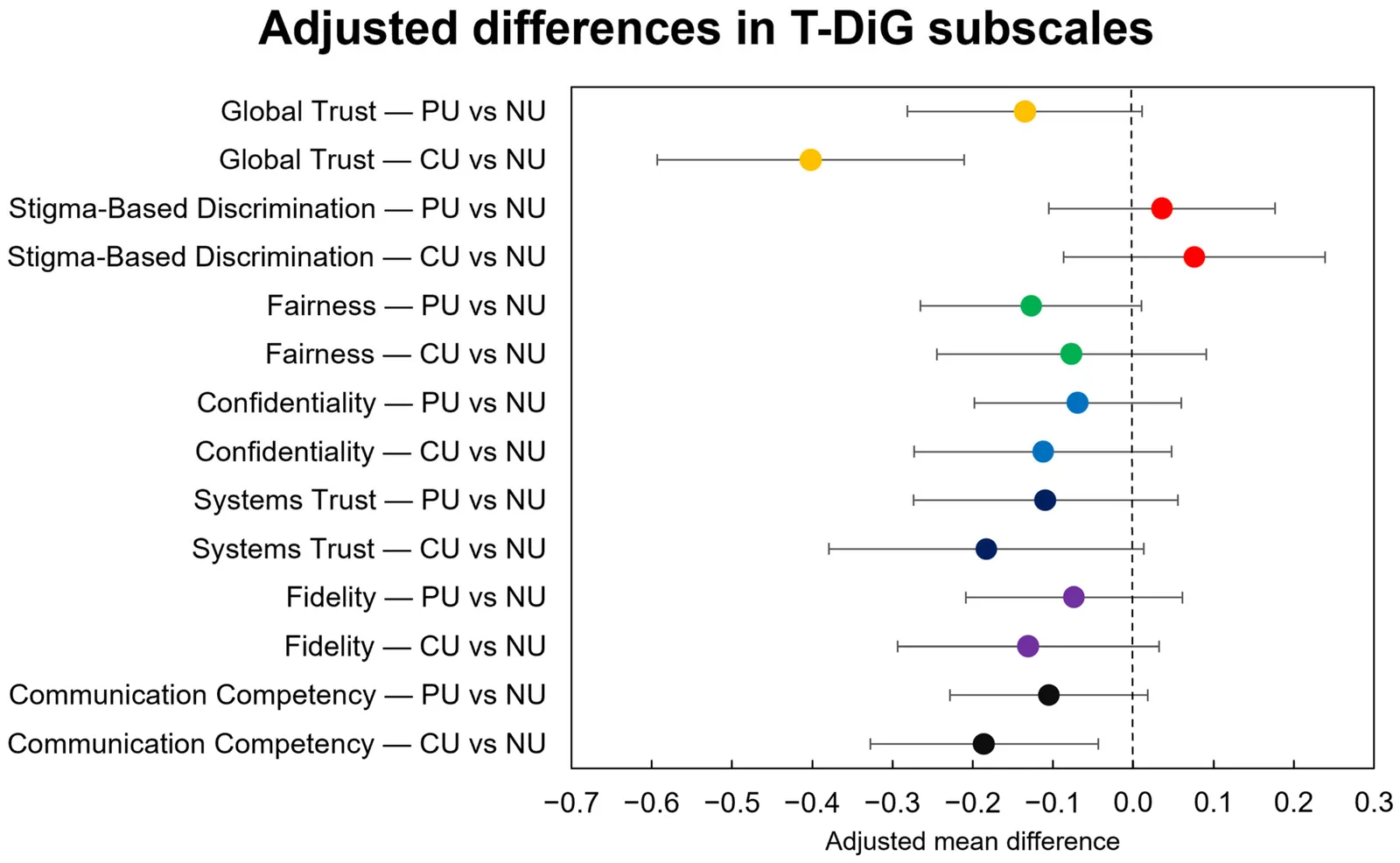
Adjusted mean differences (aMDs) and 95% confidence intervals for T-DiG subscale scores according to product-use status. CUs—current users; PUs—past users; NUs—never-users. Different colors added for better differentiation of the analyzed categories.

In adjusted linear models, product-use status was associated with communication competency (overall *p* = 0.031) and global trust (*p* < 0.001), but not with the other five subscales (all *p* ≥ 0.162). Compared with NUs, CUs had lower adjusted communication competency (aMD = −0.19, 95% CI −0.33 to −0.04; *p* = 0.010) and global trust scores (aMD = −0.40, 95% CI −0.59 to −0.21; *p* < 0.001). The corresponding PU-versus-NU estimates were not statistically significant. The results are presented in Table 6 and Figure 4.

### 3.3. Communication with Physician About Preparations

Unadjusted response distributions differed by product-use status for the first two communication items, but not for the third or fourth item (Table 7). NUs more often than CUs or PUs declared they would first discuss this issue with their doctor. They were also more likely than CUs or PUs to freely clarify any concerns with their doctor. No statistically significant differences were found on perceived doctor availability for a conversation or in willingness to have an open conversation with the doctor about current mental health issues.

**Figure 5:**
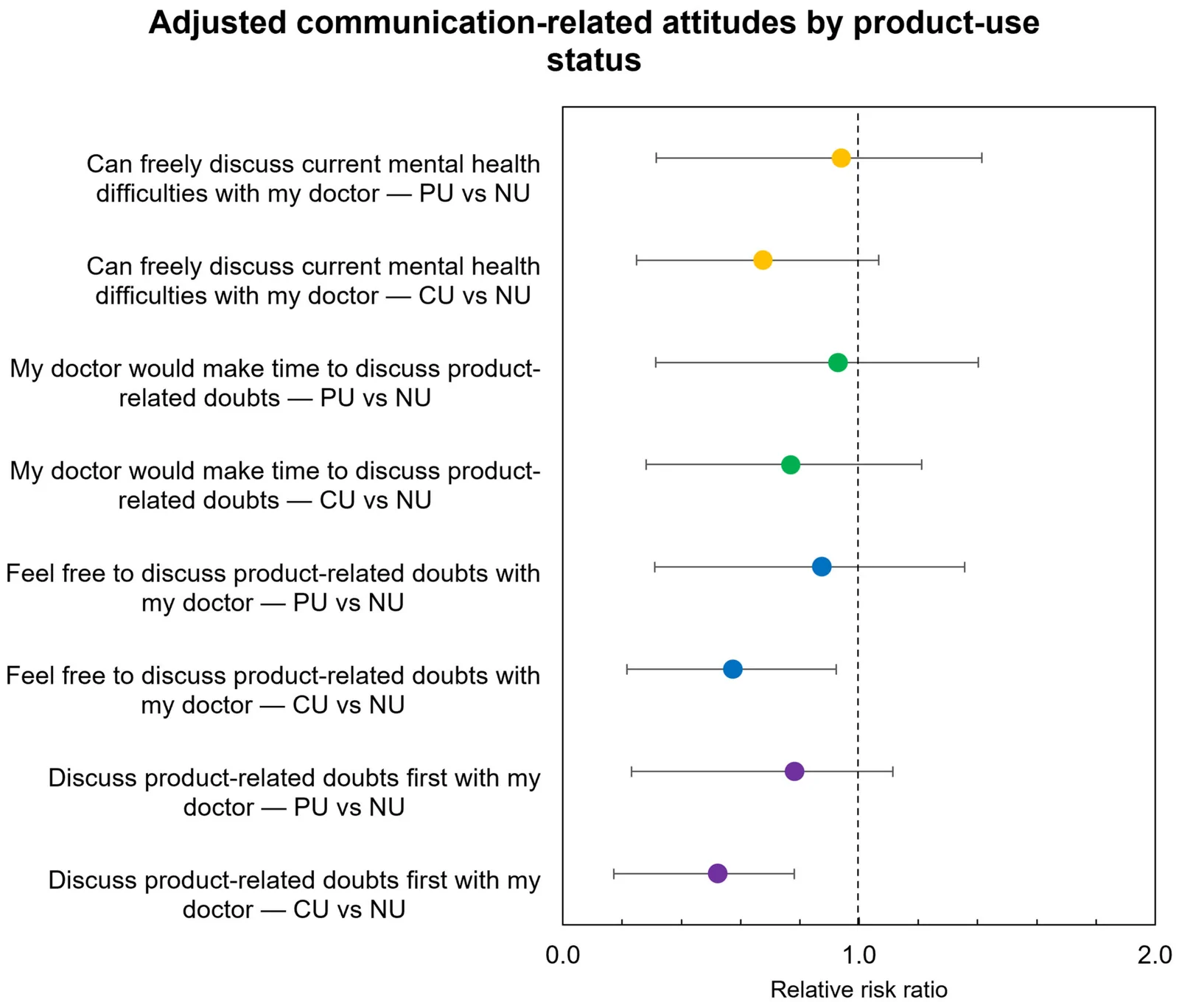
Adjusted relative risk ratios (RRRs) with 95% confidence intervals for responding “yes” versus “no” to communication-related items according to product-use status. NUs served as the reference group. CUs—current users; PUs—past users; NUs—never-users. Different colors added for better differentiation of the analyzed categories.

In adjusted multinomial models, the overall group effect remained significant for the first item (*p* < 0.001) and second item (*p* = 0.032), whereas the third (*p* = 0.809) and fourth (*p* = 0.113) were not significant (Table A4 and Figure 5).

Across the unadjusted categorical analyses, effect sizes were generally small (Cramér’s V ≤ 0.236), and several initially significant associations attenuated after covariate adjustment. Therefore, the observed differences should be interpreted as modest group-level differences rather than as clearly separated profiles of users and non-users. Statistical significance should not be equated with large or clinically decisive group differences.

## 4. Discussion

The adjusted analyses indicated that product-use status was associated with selected, rather than uniformly different, hypothetical coping preferences. The strongest association concerned future use of a mood- or stress-relief preparation: both CUs and PUs had higher adjusted odds than NUs. Product-use status was also associated with psychotherapy preference overall, with PUs showing higher adjusted odds than NUs. The unadjusted differences in psychiatric consultation and conversation with a close person were no longer statistically significant after adjustment, and no adjusted group effect was observed for GP consultation. These findings do not support a simple conclusion that people with a history of product use independently prefer either formal or informal mental healthcare. Sample composition and recruitment mode explained part of the crude group differences.

Several explanations may account for this pattern, but they should be regarded as hypotheses rather than conclusions. One possible factor is unmeasured symptom severity. Walters et al. showed that individuals with mild-to-moderate psychological distress tend to prefer informal, human-contact-based forms of help, whereas those with severe distress are more likely to favor formal treatment options, such as psychotherapy or pharmacotherapy [40]. It is therefore possible that some users experienced a greater burden of mood- or stress-related symptoms, which may have prompted both the use of mood- or stress-relief products and greater openness to professional support. CAM use was also more common among individuals with self-diagnosis [41]. However, this interpretation should be treated with caution, as psychopathological symptoms were not directly assessed in the present study. This explanation should be examined in future research.

At the same time, a previous analysis conducted in the same sample showed that the main reason NUs did not use these products was that they coped with depressed mood or stress in other ways, rather than because they did not experience such problems [39]. This suggests that differences between users and NUs may reflect not only symptom burden, but also different coping repertoires or preferences for managing psychological distress. Moreover, previous population-based research has shown that individuals using complementary approaches for anxiety or depression often do so alongside rather than instead of conventional healthcare [41]. Taken together, these findings suggest that the use of DSs and OTC medications for mood improvement or stress relief should not necessarily be interpreted as avoidance of formal care. Rather, they may reflect a broader repertoire of coping responses among individuals actively attempting to manage depressed mood or stress.

Notably, PUs, but not CUs, were more likely to indicate psychotherapy as a preferred future strategy. This may suggest that previous use of such products does not consolidate reliance on self-management. However, because the study was cross-sectional and CUs and PUs did not differ significantly in previous professional help-seeking, this result should not be interpreted as evidence of a temporal transition from product use to professional care. Rather, the possibility that coping preferences may change over time should be treated as a hypothesis for future longitudinal research. Future studies should include a direct assessment of psychopathological symptoms to clarify whether the observed differences reflect variation in symptom burden, coping preferences, or other health-related attitudes.

The present findings suggest that users and NUs differ in their level of trust in physicians, with users reporting lower scores on several subscales of T-DiG. This is broadly consistent with part of the literature on CAM, in which lower trust in conventional healthcare or healthcare professionals has been associated with greater CAM use [42,43]. However, this relationship should not be interpreted as a simple rejection of conventional medicine. Adjustment also narrowed the trust findings. Independent group differences remained for communication competency and global trust, with CUs reporting lower scores than NUs, whereas differences on the other T-DiG domains were not statistically significant after adjustment. A classic population-based study found that dissatisfaction with conventional medicine did not predict alternative medicine use, suggesting that CAM use may also reflect broader health beliefs, preferences, or values [44]. In the mental health context, complementary approaches have been shown to be used alongside rather than instead of conventional care for anxiety and depression, and cross-national data indicate that CAM contacts among people with mental disorders often occur in those also receiving conventional healthcare [41,45].

CUs also appeared less open to discussing the use of DSs and OTC medications for mood improvement or stress relief with physicians. Compared with NUs, they were less likely to identify their doctor as the first point of contact for such concerns and reported lower comfort in raising them during consultations. Systematic review evidence suggests that disclosure of CAM use to medical providers is often incomplete, particularly for biologically based products such as herbal preparations and DSs. Commonly reported reasons for non-disclosure include lack of physician inquiry, perceived clinical irrelevance, fear of disapproval, lack of time, the belief that such products are safe, and doubts about physicians’ knowledge of complementary medicine [37,38]. Previous analysis of the same sample confirmed these findings and identified considering this information clinically irrelevant and fear of negative judgement as the main reasons for non-disclosure [39]. Although the present data do not allow conclusions about the direction of the relationship between trust and disclosure-related attitudes, they suggest that lower trust in physicians and reduced openness to discussing product use may coexist among users of DSs and OTC medications for mood improvement or stress relief. This may be clinically relevant, as undisclosed use of such preparations may limit physicians’ ability to identify potential interactions, assess treatment safety, and provide appropriate guidance.

Help-seeking preferences may be shaped not only by trust, but also by perceived need, symptom burden, previous treatment experiences, expected accessibility, and the anticipated route of care [46,47,48]. These factors were not assessed in the present study. Therefore, the observed pattern should not be interpreted as contradictory evidence, but rather as an indication that product use, trust in physicians, and willingness to seek mental healthcare may be shaped by different determinants. Future studies should assess previous treatment experiences, satisfaction with care, perceived access to services, and symptom severity to clarify these relationships.

Overall, psychotherapy and psychiatric consultation were the most frequently endorsed future strategies for managing depressed mood or stress. Although this preference may reflect openness to evidence-based forms of mental healthcare, it also draws attention to the capacity of the healthcare system to meet such needs, particularly in the context of workforce shortages and limited availability of specialist services in Poland. By contrast, GP consultation was selected by 19.4% of participants and did not differ significantly by product-use status after adjustment. Because the item asked about a hypothetical situation with unlimited resources, this result is not evidence of actual underuse of primary care. It does, however, indicate that primary care was less salient than psychotherapy or psychiatric consultation in the imagined coping scenario. Contemporary integrated-care models emphasize the role of primary care in the early identification, assessment, and initial management of common mental health problems, and integrated mental health services in primary care are increasingly viewed as a way to improve access and continuity of care [49,50,51,52,53]. Patients’ perceptions of physicians also warrant attention. A considerable part of the respondents did not believe that their physician would have sufficient time to discuss the use of DSs or OTC medications during consultations. This perception is consistent with broader literature showing that consultation time is often limited and may constrain effective patient–physician communication [54,55].

For clinicians, the practical implication is not to treat self-management as inherently opposed to formal care. Qualitative research among people recovering from mood and anxiety disorders suggests that self-management may include a broad range of clinical, functional, physical, existential, and social strategies that support recovery and everyday functioning [15,56,57,58,59]. However, when self-management involves DSs or OTC medications, its potential value depends on safe and informed use, including awareness of possible interactions and communication with healthcare professionals. From a clinical perspective, the findings seem to support routine, non-judgmental inquiry about DSs and OTC medications for mood improvement or stress relief, particularly because spontaneous disclosure cannot be assumed [38,39,41,60]. At the same time, clinicians should not infer from product use alone that a patient is unwilling to consider psychotherapy, psychiatric care, or other professional support.

This study has several strengths, including the relatively large sample, the distinction between current, past, and never-users, and the simultaneous examination of coping preferences, trust in physicians, and communication-related attitudes in a context that has not previously been systematically studied.

However, several limitations should be acknowledged. The cross-sectional design precludes conclusions about causality or temporal ordering. All data were self-reported and may be subject to recall and social desirability bias. This is particularly important in the context of depressive disorders and other mental health problems. The present study did not assess psychopathology using validated clinical instruments or clinical interviews. Therefore, the findings should only be interpreted as referring to self-perceived depressed mood and stress-related difficulties. Additionally, preferred coping strategies were assessed under a hypothetical assumption of unlimited resources and may not reflect actual help-seeking behavior. Importantly, psychopathological symptoms, prior treatment experiences, and other factors that may shape both product use and help-seeking preferences were not directly assessed. Another important limitation concerns the use of an author-translated Polish adaptation of the T-DiG scale, which has not undergone formal linguistic, cultural, or psychometric validation. Cronbach’s alpha reflects internal consistency only and does not establish construct validity [35]. Finally, the sample was predominantly female, with women accounting for 80.6% of respondents. This constitutes an important limitation and may reduce the generalizability of the findings. This imbalance may partly reflect both gender differences in dietary supplement-related behaviors and differential participation in online health surveys [61,62,63]. Another important limitation concerns the recruitment strategy. Online recruitment yielded a younger, predominantly female sample with a higher proportion of participants with a medical or health science background, whereas in-person recruitment included older respondents and a higher proportion of NUs. Although these characteristics and recruitment mode were included as control variables, residual and unmeasured confounding remains possible. Therefore, the sample should not be considered representative of the general Polish population. As a result, the observed prevalence of product use, coping preferences, trust-related scores, and communication attitudes should be interpreted with caution. Because no names, email addresses, or other personal identifiers were collected, repeated online participation could not be definitively identified or ruled out. This limitation resulted from the decision to preserve participant anonymity and avoid collecting sensitive identifying information.

Future research should use prospective or longitudinal designs that measure actual disclosure and healthcare use, incorporate validated measures of depression, anxiety, and perceived stress together with diagnoses and treatment history, and formally validate a Polish T-DiG translation before confirmatory trust analyses. More representative and balanced samples, studies in other healthcare settings or countries, and sufficiently powered product-specific analyses separating dietary supplements, botanical products, and OTC medications would help determine which associations are robust across populations and product categories.

## 5. Conclusions

In this exploratory cross-sectional sample, current, past, and never-users of DSs and OTC medications for mood improvement or stress relief showed outcome-specific associations after covariate adjustment. Product-use status was most strongly associated with stated future product use. CUs also had lower adjusted communication competency and global trust scores than NUs and were less likely to report willingness to discuss potential product-related doubts first with their doctor. Adjusted overall associations with psychiatric consultation, conversation with a close person, and GP consultation were not statistically significant.

These findings do not support a simple interpretation of DS or OTC medication use for mood improvement or stress relief as reluctance to engage with formal mental healthcare. Routine, non-judgmental inquiry about such products may help improve patient–physician communication and support safer care. Further studies should include direct assessment of psychopathological symptoms and explore how symptom burden, health-related attitudes, prior treatment experiences, and trust in physicians shape coping choices and disclosure behaviors.

## Figures and Tables

**Table 1:** Sociodemographic characteristics of the study sample.

Characteristic		*n*	%
Gender	female	643	80.6
	male	149	18.7
	other/prefer not to say	6	0.8
Age group	18–24 years	263	33.0
	25–39 years	274	34.3
	40–59 years	205	25.7
	60–79 years	51	6.4
	≥80 years	5	0.6
Education	primary	8	1.0
	lower secondary	2	0.3
	vocational	24	3.0
	secondary	282	35.3
	higher	482	60.4
Medical or health science background	yes	343	43.0
	no	455	57.0
Use of DSs or OTC medications for mood improvement or stress relief	current users (CUs)	184	23.1
	past users (PUs)	297	37.2
	never-users (NUs)	317	39.7

**Table 2:** Preferred hypothetical future coping strategies among current users (CUs), past users (PUs), and never-users (NUs) of DSs or OTC medications for mood improvement and stress relief—unadjusted comparisons.

Coping Strategy	CUs (*n* = 184)		PUs (*n* = 297)		NUs (*n* = 317)		χ^2^	*p*	V
	*n*	%	*n*	%	*n*	%			
Psychotherapy	106	57.6	213	71.7	175	55.2	19.599	**<0.001**	0.157
Psychiatric consultation	105	57.1	194	65.3	159	50.2	14.426	**<0.001**	0.134
Conversation with a close person	92	50.0	161	54.2	202	63.7	10.468	**0.005**	0.115
DSs or OTC medications	66	35.9	61	20.5	35	11.0	44.375	**<0.001**	0.236
GP consultation	37	20.1	49	16.5	69	21.8	2.791	0.248	0.059

CUs—current users; PUs—past users; NUs—never-users; DSs—dietary supplement; OTC—over-the-counter; GP—general practitioner.

**Table 3:** Adjusted associations between product-use status and hypothetical future coping preferences.

Coping Strategy	CUs (*n* = 184) *n* (%)	PUs (*n* = 297) *n* (%)	NUs (*n* = 317) *n* (%)	Overall *p*	CUs vs. NUs aOR (95% CI); *p*	PUs vs. NUs aOR (95% CI); *p*
Psychotherapy	106 (57.6)	213 (71.7)	175 (55.2)	**0.018**	0.86 (0.57–1.28); *p* = 0.457	**1.50 (1.04–2.17); *p* = 0.031**
Psychiatric consultation	105 (57.1)	194 (65.3)	159 (50.2)	0.057	1.05 (0.71–1.55); *p* = 0.805	**1.50 (1.05–2.12); *p* = 0.024**
Conversation with a close person	92 (50.0)	161 (54.2)	202 (63.7)	0.105	0.68 (0.46–1.01); *p* = 0.056	0.75 (0.53–1.05); *p* = 0.095
Future DS/OTC use	66 (35.9)	61 (20.5)	35 (11.0)	**<0.001**	**5.60 (3.44–9.14); *p* < 0.001**	**2.44 (1.52–3.93); *p* < 0.001**
GP consultation	37 (20.1)	49 (16.5)	69 (21.8)	0.285	0.87 (0.55–1.38); *p* = 0.566	0.71 (0.47–1.09); *p* = 0.115

CUs—current users; PUs—past users; NUs—never-users; aOR—adjusted odds ratio; CI—confidence interval; DSs—dietary supplement; OTC—over-the-counter; GP—general practitioner.

**Table 4:** Internal consistency and descriptive statistics of the Polish T-DiG subscales.

Subscale	N	Cronbach’s α	Mean	SD
Communication competency	745	0.81	3.10	0.78
Fidelity	738	0.81	3.13	0.85
Systems trust	743	0.84	2.65	1.03
Confidentiality	740	0.84	3.87	0.84
Fairness	739	0.89	3.35	0.85
Stigma-based discrimination	736	0.79	3.34	0.85
Global trust	740	0.96	3.69	0.95

N—number; SD—standard deviation.

**Table 5:** Trust-related T-DiG subscale scores according to product-use status—unadjusted comparisons.

Subscale	CUs Mean (SD) [*n*]	PUs Mean (SD) [*n*]	NUs Mean (SD) [*n*]	H	*p*
Communication competency	2.97 (0.76) [176]	3.08 (0.79) [280]	3.20 (0.77) [289]	11.30	**0.004**
Fidelity	3.06 (0.89) [173]	3.15 (0.81) [280]	3.14 (0.87) [285]	1.46	0.482
Systems trust	2.52 (1.04) [173]	2.63 (1.01) [283]	2.74 (1.05) [287]	5.46	0.065
Confidentiality	3.77 (0.88) [171]	3.87 (0.82) [284]	3.92 (0.83) [285]	3.45	0.179
Fairness	3.32 (0.89) [171]	3.29 (0.81) [284]	3.43 (0.85) [284]	5.42	0.067
Stigma-based discrimination	3.38 (0.90) [170]	3.34 (0.87) [281]	3.32 (0.80) [285]	0.46	0.793
Global trust	3.42 (1.02) [172]	3.72 (0.87) [283]	3.83 (0.94) [285]	20.84	**<0.001**

CUs—current users; PUs—past users; NUs—never-users; H—Kruskal-Wallis test statistic; SD—standard deviation.

**Table 6:** Adjusted associations between product-use status and T-DiG subscale scores.

Subscale	CUs Mean (SD) [*n*]	PUs Mean (SD) [*n*]	NUs Mean (SD) [*n*]	Overall *p*	CUs vs. NUs aMD (95% CI); *p*	PUs vs. NUs aMD (95% CI); *p*
Communication competency	2.97 (0.76) [176]	3.08 (0.79) [280]	3.20 (0.77) [289]	**0.031**	**−0.19 (−0.33 to −0.04); *p* = 0.010**	−0.11 (−0.23 to 0.02); *p* = 0.093
Fidelity	3.06 (0.89) [173]	3.15 (0.81) [280]	3.14 (0.87) [285]	0.261	−0.13 (−0.29 to 0.03); *p* = 0.116	−0.07 (−0.21 to 0.06); *p* = 0.285
Systems trust	2.52 (1.04) [173]	2.63 (1.01) [283]	2.74 (1.05) [287]	0.162	−0.18 (−0.38 to 0.01); *p* = 0.068	−0.11 (−0.27 to 0.06); *p* = 0.193
Confidentiality	3.77 (0.88) [171]	3.87 (0.82) [284]	3.92 (0.83) [285]	0.336	−0.11 (−0.27 to 0.05); *p* = 0.169	−0.07 (−0.20 to 0.06); *p* = 0.294
Fairness	3.32 (0.89) [171]	3.29 (0.81) [284]	3.43 (0.85) [284]	0.191	−0.08 (−0.24 to 0.09); *p* = 0.368	−0.13 (−0.27 to 0.01); *p* = 0.069
Stigma-based discrimination	3.38 (0.90) [170]	3.34 (0.87) [281]	3.32 (0.80) [285]	0.650	0.08 (−0.09 to 0.24); *p* = 0.360	0.04 (−0.11 to 0.18); *p* = 0.621
Global trust	3.42 (1.02) [172]	3.72 (0.87) [283]	3.83 (0.94) [285]	**<0.001**	**−0.40 (−0.59 to −0.21); *p* < 0.001**	−0.14 (−0.28 to 0.01); *p* = 0.070

CUs—current users; PUs—past users; NUs—never-users; CI—confidence interval; aMD—adjusted mean difference in subscale score relative to NUs.

**Table 7:** Patient–physician communication regarding preparations for mood improvement or stress relief.

Item	CUs	PUs	NUs	χ^2^	*p*	V
	Yes/No/No Opinion; *n* (%)	Yes/No/No Opinion; *n* (%)	Yes/No/No Opinion; *n* (%)			
If I had any doubts about using preparations for mood improvement or stress relief, I would first discuss this with my doctor	73 (40.1)/ 87 (47.8)/ 22 (12.1)	146 (49.7)/ 118 (40.1)/ 30 (10.2)	172 (58.1)/ 111 (37.5)/ 13 (4.4)	20.02	**<0.001**	0.114
If I had any doubts about using preparations for mood improvement or stress relief, I would feel free to discuss this with my doctor	103 (56.9)/ 45 (24.9)/ 33 (18.2)	206 (69.8)/ 55 (18.6)/ 34 (11.5)	214 (72.1)/ 51 (17.2)/ 32 (10.8)	13.24	**0.010**	0.093
If I had doubts about using such preparations and wanted to discuss this with my doctor, they would make time for it	84 (46.2)/ 52 (28.6)/ 46 (25.3)	145 (49.2)/ 73 (24.7)/ 77 (26.1)	155 (52.2)/ 69 (23.2)/ 73 (24.6)	2.29	0.683	0.038
I can talk freely with my doctor about my current mental health difficulties, such as low mood or stress	87 (47.8)/ 48 (26.4)/ 47 (25.8)	171 (58.0)/ 67 (22.7)/ 57 (19.3)	176 (59.3)/ 69 (23.2)/ 52 (17.5)	7.68	0.104	0.070

CUs—current users; PUs—past users; NUs—never-users.

## Data Availability

The data, analysis code, and questionnaire materials are available from the corresponding authors upon reasonable request, subject to applicable ethical and data-protection conditions.

## Abbreviations

The following abbreviations are used in this manuscript.

aMD	adjusted mean difference
aOR	adjusted odds ratio
CAM	complementary and alternative medicine
CUs	current users
CI	confidence interval
DSs	dietary supplements
GP	general practitioner
H1	hypothesis 1
H2	hypothesis 2
H3	hypothesis 3
NUs	never-users
OTC	over-the-counter medication
PUs	past users
RRR	relative risk ratio
SD	standard deviation
T-DiG	Trust in Doctors in General
T-MD	Trust in My Doctor

## Appendix A

**Table A1:** Items from the Trust in Doctors in General scale with Polish translations (translated by authors for the purpose of the study, not formally validated).

Subscale	Original Item	Polish Translation
**Communication competency**	Doctors have good judgement	Lekarze właściwie oceniają sytuację swoich pacjentów
	Doctors explain the benefits and risks of treatments to patients	Lekarze wyjaśniają pacjentom korzyści i zagrożenia związane z procesem leczenia
	Doctors listen to patients	Lekarze słuchają pacjentów
	Doctors believe patients when they say something is wrong	Lekarze wierzą pacjentom, kiedy ci zgłaszają jakieś dolegliwości
	Doctors follow up with patients when needed	Lekarze sprawdzają stan swoich pacjentów po wizycie, jeśli jest taka potrzeba
**Fidelity**	Doctors put making money above patient needs	Dla lekarzy zarabianie pieniędzy jest ważniejsze od potrzeb pacjentów
	Doctors recommend expensive treatments to make money	Lekarze zalecają kosztowne kuracje, aby się dorobić
	Doctors hide mistakes	Lekarze ukrywają swoje błędy
	Doctors might experiment on patients without their knowledge	Lekarze mogą eksperymentować na pacjentach bez ich wiedzy
	Doctors rush through appointments	Lekarze przeprowadzają wizyty pośpiesznie
**Systems trust**	Doctors are held accountable if they make a mistake	Lekarze są pociągani do odpowiedzialności, jeśli popełnią błąd
	Doctors are held accountable if they treat patients unfairly	Lekarze są pociągani do odpowiedzialności, jeśli traktują pacjentów niesprawiedliwie
	Doctors are held accountable if they discriminate against patients	Lekarze są pociągani do odpowiedzialności, jeśli dyskryminują pacjentów
**Confidentiality**	Doctors keep medical records private	Lekarze zachowują poufność dokumentacji medycznej
	Doctors use secure systems to store medical records	Lekarze przechowują dokumentację medyczną pacjentów w bezpieczny sposób
	Doctors respect patient privacy	Lekarze szanują prywatność pacjentów
**Fairness**	Doctors treat patients fairly, regardless of their ability to pay	Lekarze traktują pacjentów równo niezależnie od ich możliwości finansowych
	Doctors treat patients of all races and ethnicities fairly	Lekarze traktują pacjentów wszystkich ras i grup etnicznych w równy sposób
	Doctors treat patients fairly, regardless of their gender (e.g., male, female, or nonbinary)	Lekarze traktują pacjentów równo niezależnie od ich płci
	Doctors would treat me fairly, regardless of my sexual orientation (e.g., straight, gay, lesbian, or bisexual)	Lekarze traktują pacjentów równo bez względu na ich orientację seksualną (np. heteroseksualną, homoseksualną, biseksualną)
	Doctors treat patients fairly, regardless of their weight	Lekarze traktują pacjentów równo bez względu na masę ciała
	Doctors treat patients fairly, regardless of their religion	Lekarze traktują pacjentów równo bez względu na religię
	Doctors treat patients fairly, regardless of their education level	Lekarze traktują pacjentów równo bez względu na poziom wykształcenia
**Stigma-based discrimination**	Doctors treat patients with a history of mental illness unfairly	Lekarze traktują niesprawiedliwie pacjentów z historią choroby psychicznej
	Doctors treat patients diagnosed with HIV unfairly	Lekarze traktują niesprawiedliwie pacjentów z HIV
	Doctors treat patients who abuse drugs unfairly	Lekarze traktują niesprawiedliwie pacjentów, którzy nadużywają leków
**Global trust**	All things considered, I trust doctors	Ogólnie rzecz biorąc, wierzę lekarzom
	I put my trust in doctors	Darzę lekarzy swoim zaufaniem
	Doctors are trustworthy	Lekarze to ludzie godni zaufania

**Table A2:** Comparison of sociodemographic characteristics and product-use status by recruitment mode.

Characteristic		Online (*n* = 636), *n* (%)	In-Person (*n* = 162), *n* (%)	χ^2^	*p*	V
Gender				35.45	**<0.001**	0.211
	female	539 (84.7)	104 (64.2)			
	male	94 (14.8)	55 (34.0)			
	other/prefer not to say	3 (0.5)	3 (1.9)			
Age group				259.11	**<0.001**	0.570
	18–24 years	257 (40.4)	6 (3.7)			
	25–39 years	248 (39.0)	26 (16.0)			
	40–59 years	121 (19.0)	84 (51.9)			
	≥60 years	10 (1.6)	46 (28.4)			
Education				100.63	**<0.001**	0.355
	primary/lower secondary/vocational	5 (0.8)	29 (17.9)			
	secondary	248 (39.0)	34 (21.0)			
	higher	383 (60.2)	99 (61.1)			
Medical or health science background				108.64	**<0.001**	0.369
	yes	332 (52.2)	11 (6.8)			
	no	304 (47.8)	151 (93.2)			
Use of DSs or OTC medications for mood improvement or stress relief				15.41	**<0.001**	0.139
	current users (CUs)	160 (25.2)	24 (14.8)			
	past users (PUs)	244 (38.4)	53 (32.7)			
	never-users (NUs)	232 (36.5)	85 (52.5)			

**Table A3:** Complete response distributions for previous use of professional support for low mood or stress among current and past users of dietary supplements or OTC medications for mood improvement or stress relief.

Item	Answer	CUs (*n* = 184), *n* (%)	PUs (*n* = 297), *n* (%)	χ^2^	*p*	V (95% CI)
Help from a doctor	yes	99 (53.8)	182 (61.3)	2.709	0.258	0.075 (0.000–0.155)
	no	80 (43.5)	107 (36.0)			
	prefer not to answer	5 (2.7)	8 (2.7)			
Help from a psychologist	yes	97 (52.7)	184 (62.0)	4.402	0.111	0.096 (0.000–0.177)
	no	84 (45.7)	107 (36.0)			
	prefer not to answer	3 (1.6)	6 (2.0)			

CUs—current users; PUs—past users; CI—confidence interval.

**Table A4:** Patient–physician communication regarding mood- and stress-relief preparations by product-use status.

Item	Response Contrast	Overall *p*	CUs vs. NUs RRR (95% CI); *p*	PUs vs. NUs RRR (95% CI); *p*
If I had any doubts about using preparations for mood improvement or stress relief, I would first discuss this with my doctor	Yes vs. No	**<0.001**	0.52 (0.35–0.78); ***p* = 0.001**	0.78 (0.55–1.11); *p* = 0.173
	No opinion vs. No	—	2.00 (0.95–4.23); *p* = 0.070	2.11 (1.03–4.32); *p* = 0.040
If I had any doubts about using preparations for mood improvement or stress relief, I would feel free to discuss this with my doctor	Yes vs. No	**0.032**	0.57 (0.36–0.92); ***p* = 0.022**	0.88 (0.56–1.36); *p* = 0.551
	No opinion vs. No	—	1.18 (0.62–2.24); *p* = 0.614	0.96 (0.51–1.81); *p* = 0.905
If I had doubts about using such preparations and wanted to discuss this with my doctor, they would make time for it	Yes vs. No	0.809	0.77 (0.49–1.21); *p* = 0.258	0.93 (0.62–1.40); *p* = 0.730
	No opinion vs. No	—	0.80 (0.48–1.35); *p* = 0.408	1.02 (0.64–1.63); *p* = 0.948
I can talk freely with my doctor about my current mental health difficulties, such as low mood or stress	Yes vs. No	0.113	0.68 (0.43–1.07); *p* = 0.092	0.94 (0.62–1.42); *p* = 0.768
	No opinion vs. No	—	1.26 (0.72–2.20); *p* = 0.411	1.17 (0.69–1.98); *p* = 0.553

CUs—current users; PUs—past users; NUs—never-users; RRR—relative risk ratio; CI—confidence interval.
